# The Betting Odds Rating System: Using soccer forecasts to forecast soccer

**DOI:** 10.1371/journal.pone.0198668

**Published:** 2018-06-05

**Authors:** Fabian Wunderlich, Daniel Memmert

**Affiliations:** Institute of Training and Computer Science in Sport, German Sport University Cologne, Cologne, Germany; Queen Mary University of London, UNITED KINGDOM

## Abstract

Betting odds are frequently found to outperform mathematical models in sports related forecasting tasks, however the factors contributing to betting odds are not fully traceable and in contrast to rating-based forecasts no straightforward measure of team-specific quality is deducible from the betting odds. The present study investigates the approach of combining the methods of mathematical models and the information included in betting odds. A soccer forecasting model based on the well-known ELO rating system and taking advantage of betting odds as a source of information is presented. Data from almost 15.000 soccer matches (seasons 2007/2008 until 2016/2017) are used, including both domestic matches (English Premier League, German Bundesliga, Spanish Primera Division and Italian Serie A) and international matches (UEFA Champions League, UEFA Europe League). The novel betting odds based ELO model is shown to outperform classic ELO models, thus demonstrating that betting odds prior to a match contain more relevant information than the result of the match itself. It is shown how the novel model can help to gain valuable insights into the quality of soccer teams and its development over time, thus having a practical benefit in performance analysis. Moreover, it is argued that network based approaches might help in further improving rating and forecasting methods.

## Introduction

Forecasting sports events like matches or tournaments has attracted the interest of the scientific community for quite a long time. Sports events like soccer matches take place regularly and generate huge public attention. Moreover, extensive data are available and relatively easy to interpret. Due to these factors, sports (and especially soccer) turn out to be a perfect environment to study the applicability of existing forecasting methods or develop new methods to be transferred to other fields of forecasting.

Searching for the most accurate sports forecasting methods is both interesting from a scientific view and from an economic view as the huge betting market for soccer (and other sports) is providing the opportunity to win money by forecasting accurately [1]. Besides providing accurate forecasts the forecasting models can also be valuable in understanding the nature of the underlying processes [2] and, as demonstrated within this study, to gain practical insights to performance analysis in sports.

Three different tasks contribute to the complexity of approaching sports forecasts with the use of mathematical models. First, the unknown quality of a team (or player) needs to be investigated utilizing a wide and meaningful data set as well as a well-fitted mathematical model [3,4]. Second, the forecast itself (i.e. probability of a certain match or tournament outcome) needs to be derived using appropriate statistical methods such as probability models [5] or Monte Carlo simulation [2,6]. Finally, the results of the forecasts need to be tested against real data using appropriate statistical tests. We will refer to these three challenges as *rating process*, *forecasting process* and *testing process* throughout the paper.

Various sources of forecasts have been investigated in an attempt to understand forecasting processes, develop promising forecasting methods and compare their forecasting abilities. The sources can be broadly classified in four categories:

1. Human judgement, i.e. asking participants with a varying degree of knowledge to perform sports-related forecasting tasks
2. Rankings, i.e. using official rankings such as the FIFA World Ranking in soccer or the ATP ranking in tennis to derive forecasts for future matches and tournaments.
3. Mathematical models, i.e. using existing or developing novel mathematical and statistical approaches to forecast the outcomes of sports events.
4. Betting odds, i.e. using the odds offered by bookmakers and betting exchanges as a forecast of the underlying sports event.

### Human judgement

Numerous works have investigated the predictive quality of human forecasts in soccer. In general, so-called soccer experts are not able to outperform laypeople on simple soccer related forecasting tasks [7]. Moreover, most participants were outperformed by forecasts following a simple rule based on the FIFA World Ranking in the aforementioned study. Expert forecasts from tipsters published in sports journals were even shown to be outperformed by the naïve model of always selecting the home team to win [8]. However, it was shown that experts outperform laypeople in more complex forecasting tasks such as forecasting exact scores or match statistics [9].

### Rankings

The predictive character of rankings is questionable for several reasons. Rankings are usually designed to reward for success and not to make the best estimate on a future performance of a team or player. Moreover, sports rankings are simplistic and lack relevant information for the purpose of being fair and easy to understand (cf. [10]). However, rankings are found to be useful predictors in general for soccer [11], tennis [10] and basketball [12]. At the same time it is shown that betting odds [11] or mathematical models [10] are capable of outperforming these rankings in predictive tasks.

### Mathematical models

A frequently investigated and widely accepted mathematical approach in sports forecasting is the ELO rating system, which is a well-known method for ranking and rating sports teams or players. It was originally invented for and used in chess, but throughout the time it has been successfully applied to a variety of other sports including soccer (see [13,3]), tennis [14] or Australian rules football [15].

Hvattum and Arntzen [16] extended the well-known ELO rating system using logit regression models to calculate probabilities for the three match outcomes (Home/Draw/Away) from the ELO ratings. It was shown that this ELO approach was superior to models based on an ordered probit regression approach introduced by Goddard [17] but inferior to betting odds.

### Betting odds

Betting odds can be seen as an aggregated expert opinion reflecting both the judgement of bookmakers and the betting behavior of bettors. However, it is a completely different form of expert opinion compared to studies where experts are asked to perform forecasting tasks in an experimental environment. Whereas those experts usually do not have to fear negative consequences from inaccurate forecasts, offering inaccurate odds will have serious financial consequences for bookmakers. This could be a reason why betting odds were shown to be clearly outperforming soccer tipsters publishing their forecasts in sports journals [8].

Hvattum and Arntzen [16] show that in general betting odds possess an excellent predictive quality and perform better in forecasting soccer results than various quantitative models. A consensus model based on betting odds of various bookmakers was shown to provide more accurate forecasts on the European championship 2008 in soccer than methods using the ELO rating and the FIFA World Ranking [11]. Kovalchik [14] even investigates eleven forecasting models in tennis and finds that none of it is able to outperform betting odds in forecasting singles matches.

Without denying the general predictive power of betting odds, it is worth noting that there are empirical indications on the imperfectness of betting odds as shown in [18] or in the extensively documented favorite-longshot bias (see [19] for an overview). Moreover, it is worth noting that various model based approaches were yielding positive betting returns when deducing betting strategies from the forecasts ([20–22] among others).

A major part of the aforementioned studies focuses on comparing the four different sources of forecasts or different approaches for the same source of forecast. As a wide consensus exists that betting odds have proven to be a powerful instrument in forecasting [23], betting odds are routinely used as a quality benchmark for testing the predictive quality of mathematical approaches [14]. By doing this, betting odds and mathematical models are outlined as contrary approaches for the same forecasting task, instead of mixing the power of both approaches to create new forecasting possibilities.

So far, hardly any study has tried to revert the forecasting process using existing forecasts (from betting odds) to draw conclusions about the qualities of the teams, obtain team ratings and thus contribute to the performance analysis of teams. Leitner et al. [11] pursue this strategy by using an “inverse” simulation of the European Championship in 2008 to obtain team ratings from the betting odds for the tournament. This approach especially sheds light on the differences between a team’s quality and its probability of winning a tournament (the effects of tournament draws). However, no betting odds from single matches are considered for establishing team ratings. Although the predictive quality of betting odds is frequently stated and the extensive information reflected in the odds can undisputedly be seen as an important advantage of betting odds, the question of how valuable betting odds of prior matches are for forecasting future matches has not been tackled so far.

This study extends prior research in various aspects. We present a novel model that is able to combine the advantages of mathematical approaches with the information advantage of betting odds. By design, the model is not expected to improve forecasts from betting odds, but it aims at developing a framework that enables us to investigate the transferability of prior forecasts to future forecasts, construct a rating that improves classical rating methods and thus use forecasting methods to gain improved practical insights into performance analysis. In detail, we examine the question whether betting odds known prior to a match are of higher value for forecasting purposes than the result known after the match. The rating used as an intermediate step of the forecasting model can be interpreted as a reversal of the forecasting process as the quality of a soccer team is deduced from prior forecasts. We use this rating to demonstrate improvements to traditional rating methods and how the information included in betting odds can effectively be extracted to be used in practical analysis, e.g. on the quality development of soccer teams. Moreover, we demonstrate how the ELO-Odds model can be used for analyzing the quality development of individual teams over time or the explanatory power of league tables. Finally, we demonstrate a lack of theoretical foundations concerning rating models that take advantage from the network structure of matches by applying match results to the ratings of uninvolved teams.

## Method

### Data

We obtained match data for 10 seasons in four of the most important European soccer leagues (namely the English Premier League, the German Bundesliga, the Spanish Primera Division and the Italian Serie A) from http://www.football-data.co.uk. For each league all seasons from 2007/2008 until 2016/2017 were considered adding up in a total data set of nearly 14,500 domestic soccer matches. Moreover, we obtained data for 10 seasons in the most important international club competitions (UEFA Champions League and UEFA Europe League) from http://www.oddsportal.com. For all seasons from 2007/2008 until 2016/2017 those matches played between participants from the four aforementioned soccer leagues were considered. Overall, more than 450 international matches were considered adding up in a total database of nearly 15,000 matches.

The models examined throughout this paper are based on the following data for each match: match date, home team, away team, home goals (full time), away goals (full time) as well as betting odds for home win, draw and away win. To avoid bookmaker-specificity and obtain a best possible reflection of the betting market, all betting odds used in the analysis are averaged based on available betting odds of various different bookmakers. Except for isolated cases, the average betting odds are based on five or more bookmakers in international matches and 20 or more bookmakers in domestic matches. The difference between international and domestic matches is due to the extent of information and level of detail available at the respective data source. The matches Cagliari vs. Roma (23.09.12) and Sassuolo vs. Pescara (28.08.16) were completely discarded from the data set as both were decided by federation decision. The final matches from Champions League and Europe League were completely excluded from the data set as these are played at a neutral location. See Table 1 for detailed information on the number of matches for each season and competition.

**Table 1:** Information on the data set used within this study.

Competition	Seasons	Number of matches	Average overround	Average theoretical bookmaker payout
English Premier League	07/08–16/17	3,800	1.065	0.939
German Bundesliga	07/08–16/17	3,060	1.060	0.944
Spanish Primera Division	07/08–16/17	3,800	1.065	0.939
Italian Serie A	07/08–16/17	3,798	1.067	0.937
UEFA Champions League	07/08–16/17	316	1.047	0.956
UEFA Europe League	07/08–16/17	157	1.054	0.949
Total	07/08–16/17	14,931	1.064	0.940

### Transferring betting odds to probabilities

Betting odds are widely used to derive forecasts as they are simply transferrable to probabilities and have proven their quality in a large number of different studies. If no bookmaker margin was contained in the betting odds, the inverse betting odds for any possible outcome of a match could be interpreted as its probability of occurring. To eliminate the bookmaker margin from the odds, i.e. ensure that the derived probabilities sum up to 100%, we applied the most widely used approach of basic normalization (see [11,24] for a more detailed explanation and S1 File for details on the calculation). This approach eliminates the overall bookmaker margin, however it can be criticized as simplifying, as it implicitly assumes that bookmaker margin is distributed proportionately across all possible outcomes of a match (e.g. home, win and draw). For a more detailed discussion on this issue, possible consequences and alternative approaches see [25,24]. Due to the reasonably small margins in our data set (average bookmaker overround of 1.064 corresponding to a theoretical payout of 94.0%) we consider the approach of basic normalization an acceptable simplification. See Table 1 and S1 File for more details on the margins.

### Rating systems

The ELO rating system is a well-known and widely used rating system that was originally invented to be used in chess, but has successfully been transferred to rate soccer teams (cf. [3]). The model is based on the idea of calculating an expected result for each match from the current rating of the participating teams. After the match the actual result is known and the ratings of both participants are adjusted accordingly. A higher difference between actual result and expected result evokes a higher adjustment made to the ratings (and vice versa). As a result, for each team a dynamic rating is obtained and is adjusted over time by every new match result that becomes observable.

### ELO-Result

Let *H*_*i*_ and *A*_*i*_ be the ELO-ratings for the home and the away team prior to a match. Then the expected result for the match is

$$e^{H}=\frac{1}{1+c^{(A_{i}-H_{i}-\omega )/d}}$$

$$e^{A}=1-e^{H}$$

where *ω* is a measure for the home advantage (in ELO-points) while *c* and *d* are freely selectable parameters that influence the scale of the rating. Within this study, we apply the usual choice of *c* = 10 and *d* = 400.

After the match the actual result *a*^*H*^ for the home team can be observed. It is set as *a*^*H*^ = 1 if the home team wins, *a*^*H*^ = 0.5 in case of a draw and *a*^*H*^ = 0 if the home team loses. The actual result for the away team consequently is *a*^*A*^ = 1 − *a*^*H*^ and the ratings for both teams are adjusted as follows:

$$H_{i+1}=H_{i}+k\;(a^{H}-e^{H})$$

$$A_{i+1}=A_{i}+k\;(a^{A}-e^{A})$$

where *k* is an adjustment factor that we will choose by calibrating. We refer to this classic ELO model as ELO-Result. See [26] and [13,3] for more information on the calculation of a classic ELO rating in chess and soccer.

### ELO-Goals

This modification of the ELO model additionally takes the goals scored by each team into account. Let *δ* be the absolute goal difference for a match. Then the parameter k is modified to be

$$k=k_{0}{\;(1+\delta )}^{\lambda }$$

Therefore, the model is able to use more information than the pure result of a match. The calculation has been adopted from [16] and the model is referred to as ELO-Goals. Note that the well-known World Football Elo Ratings published online [13,3] is also based on a calculation including the goals, however using a slightly different calculation method.

### ELO-Odds

Although betting odds have proven to possess excellent predictive qualities, they have not been used as a basis to create rankings and ratings. Surprisingly it has not been evaluated yet, how valuable betting odds from previous matches are for forecasting future soccer matches. The following model is referred to as ELO-Odds and combines the methods of ELO-rating with the information obtained from betting odds.

The calculation works similar as shown for ELO-Result, i.e. the expected result for each match is calculated from the current rating of its participants. The actual result, however, is replaced by the expected result in terms of betting odds. Let *p*_*H*_, *p*_*D*_ and *p*_*A*_ be the probabilities for home win, draw and away win obtained from the betting odds. Then the actual result as used in ELO-Result is replaced by:

$$a^{H}=p_{H}+0.5\;p_{D}$$

$$a^{A}=p_{A}+0.5\;p_{D}=1-a^{H}$$

The model aims at accessing more information than results or goals by indirectly deriving it from the betting odds. At the same time, it is a drastic restriction as throughout the calculation of the ELO-Odds ratings no match result is ever directly used. Moreover, the model uses the betting odds prior to the match as a measure for the actual result, thus only using information that was known prior to the start of the match and fully ignoring the result that is observable after the match.

### Statistical framework

To make sure this study is based on a solid framework, we make use of previous research and proven statistical methods, that are largely adopted from Hvattum and Arntzen [16]. For each of the ELO models the approach is as follows: For the full time period of data (10 seasons, 07/08–16/17) the ELO rating of each team is calculated and adjusted after each match. A home advantage of *ω* = 80 is used as found in the aforementioned paper. As a start value each team is given a rating of 1,000 points prior to the first match of the first season. To have a useful start value for promoted teams in later seasons, these teams carry on the ratings of the relegated teams. This procedure has two positive effects: First, it can be assumed that promoted teams are in general weaker than the average team in the league. Thus the ratings of the relegated teams are a more promising estimator of team quality than using an average start value for the promoted teams. Second, it has the nice side-effect that the sum of ratings stays the same over the full period of time, calculated over all teams that are currently participating in one of the four leagues.

**Fig 1:**
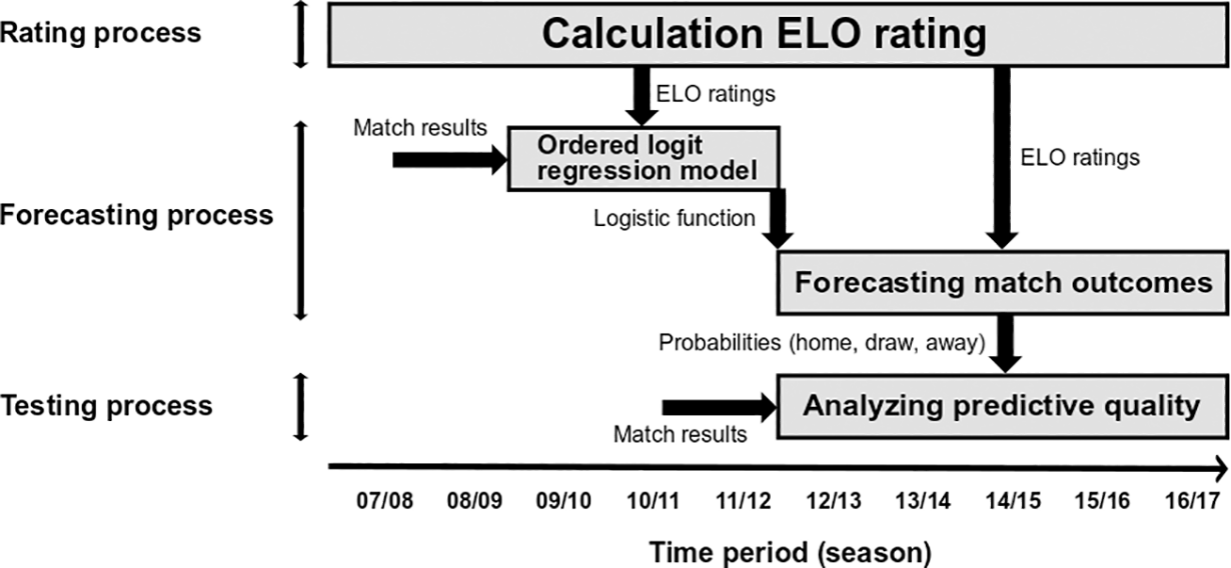
The forecasting methods and statistical framework as used within this study and largely obtained from Hvattum and Arntzen.

The first two seasons (07/08 & 08/09) solely serve as a time period to derive a useful initial rating for each team. For each match of the following three seasons (09/10–11/12) the difference between the home team’s rating and the away team’s rating is obtained. These rating differences then are taken as the single covariate of an ordered logit regression model. As a result from the regression model, logistic functions are obtained that transfer a rating difference into probabilities for home win, draw and away win. For each match of the last five seasons (12/13–16/17) these probabilities are calculated and form the forecasts of the matches. Finally, the forecasts are analyzed using the *informational loss L*_*i*_ (see [27] for a definition) as a measure of predictive quality. Please note that minimizing the informational loss is equivalent to maximizing the likelihood function. To verify whether differences regarding the loss functions of two models are significant, paired t-tests are used. See Fig 1 for a graphical representation of rating process, forecasting process and testing process.

## Results

### Parameter calibration

**Fig 2:**
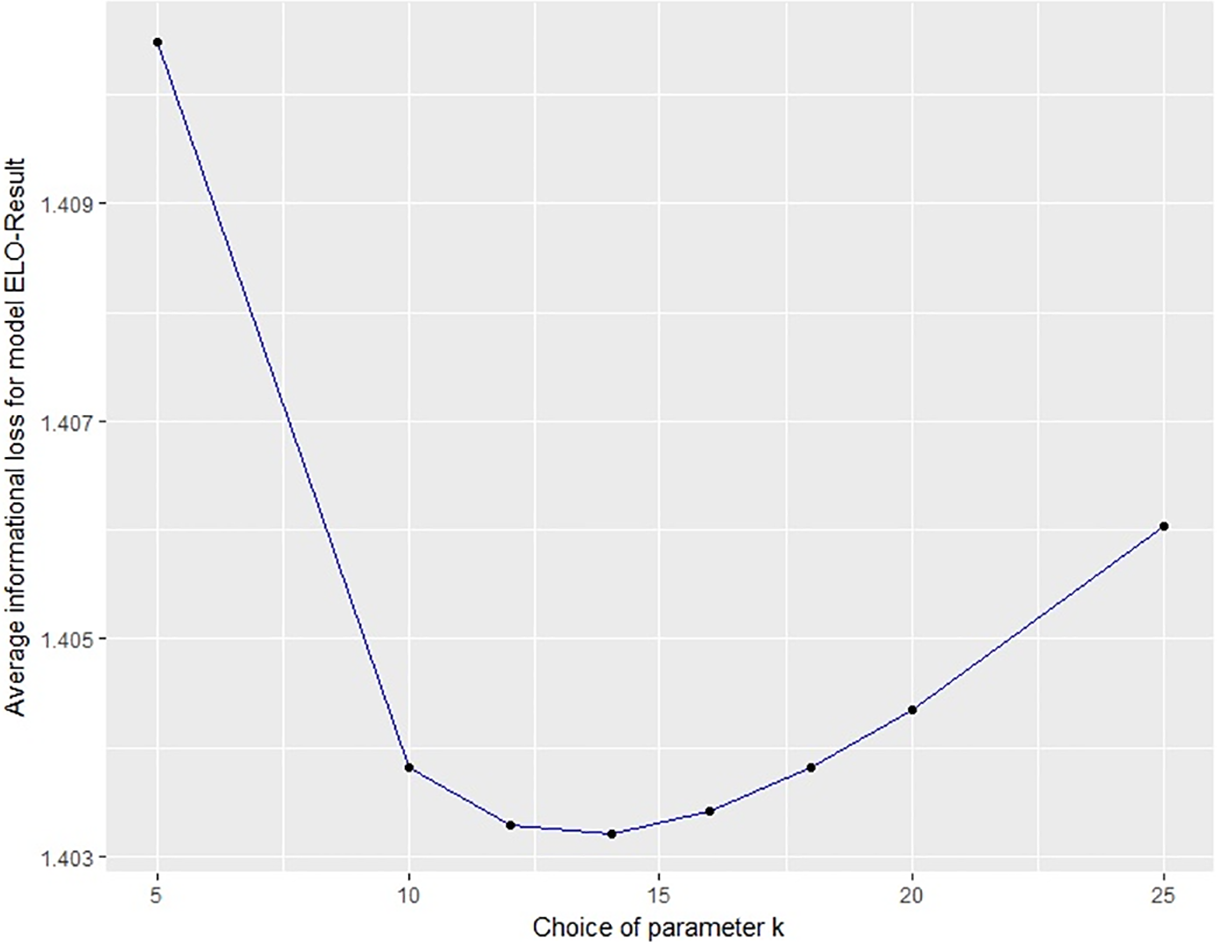
Average informational loss for various choices of the parameter k in model ELO-Result.

**Fig 3:**
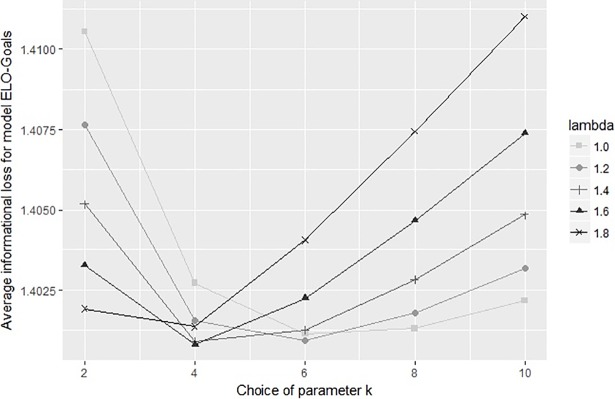
Average informational loss for various choices of the parameters k and lambda in model ELO-Goals.

**Fig 4:**
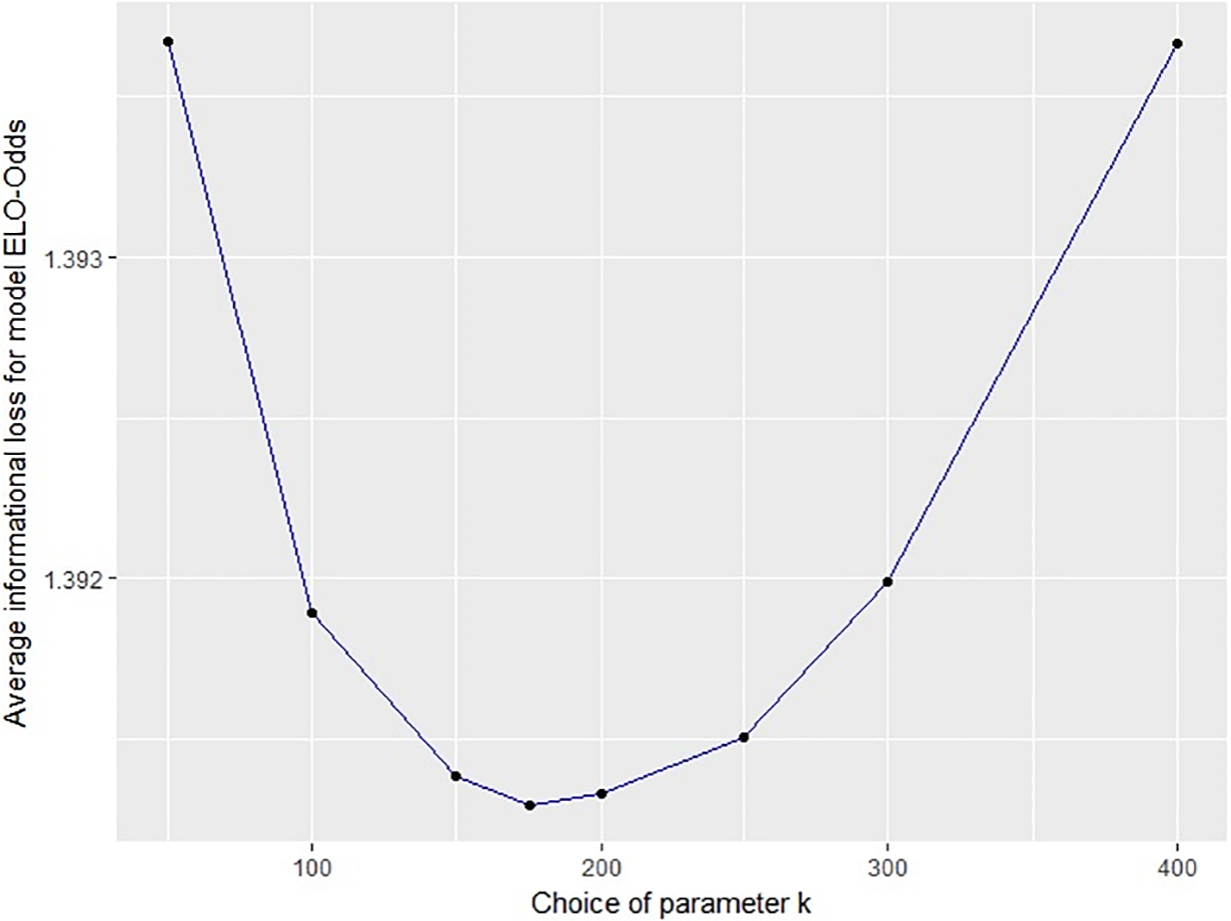
Average informational loss for various choices of the parameter k in model ELO-Odds.

The three models ELO-Result, ELO-Goals and ELO-Odds require calibration of parameters. Whereas ELO-Result and ELO-Odds require one single parameter *k*, ELO-Goals requires two parameters *k*_0_ and *λ*. Table 2 shows the informational loss when choosing different parameters for ELO-Result, ELO-Goals and ELO-Odds. The informational loss for all three models and different parameters is moreover illustrated in Fig 2, **Fig 3** and Fig 4. From the results we can choose useful parameters for the models (namely *k* = 14 for ELO-Result; *k*_0_ = 4, *λ* = 1.6 for ELO-Goals and *k* = 175 for ELO-Odds).

**Table 2:** Comparison of informational loss for different models and various parameters.

Forecasting model	Parameters	Average L_i_
Betting Odds	-	1.3795
ELO-Odds	k = 175	1.3913
ELO-Odds	k = 200	1.3913
ELO-Odds	k = 150	1.3914
ELO-Odds	k = 250	1.3915
ELO-Odds	k = 100	1.3919
ELO-Odds	k = 300	1.3920
ELO-Odds	k = 400	1.3937
ELO-Odds	k = 50	1.3937
ELO-Goals	k_0_ = 4, λ = 1.6	1.4008
ELO-Goals	k_0_ = 4, λ = 1.4	1.4009
ELO-Goals	k_0_ = 6, λ = 1.2	1.4009
ELO-Goals	k_0_ = 6, λ = 1.0	1.4011
ELO-Goals	k_0_ = 2, λ = 2.0	1.4012
ELO-Goals	k_0_ = 6, λ = 1.4	1.4013
ELO-Goals	k_0_ = 8, λ = 1.0	1.4013
ELO-Goals	k_0_ = 8, λ = 0.8	1.4013
ELO-Goals	k_0_ = 4, λ = 1.8	1.4014
ELO-Goals	k_0_ = 4, λ = 1.2	1.4016
ELO-Result	k = 14	1.4032
ELO-Result	k = 12	1.4033
ELO-Result	k = 16	1.4034
ELO-Result	k = 18	1.4038
ELO-Result	k = 10	1.4038
ELO-Result	k = 20	1.4043
ELO-Result	k = 25	1.4060
ELO-Result	k = 5	1.4105

At first glance, it is surprising that the adjustment factor *k* is more than ten times higher for ELO-Odds than for ELO-Result, but this result can be explained as follows: First, the actual results (*a*^*H*^, *a*^*A*^) in ELO-Result being either 0, 0.5 or 1 naturally deviate more from the expected result than in ELO-Odds, consequently requiring a smaller adjustment factor. Second, the actual results in ELO-Result are subject to strong influence of randomness. A higher adjustment factor does therefore evoke a too strong adaption of the latest results.

In general, using the results to choose the parameters (i.e. selecting those parameters yielding the best results) evokes a danger of overfitting the data. However, we can see that the results are not highly sensitive to the choice of the parameter(s), compared to the sensitivity of the results to the choice of the model (see next section).

### Predictive quality

Table 3 shows the major results of analyzing the predictive quality of the different forecasting methods. Betting odds are shown to have the highest predictive quality, outperforming ELO-Odds on a highly significant level. ELO-Odds in turn is outperforming ELO-Goals on a highly significant level while ELO-Goals is outperforming ELO-Result significantly. Therefore, the results of Hvattum and Arntzen [16] could be reproduced with respect to betting odds, ELO-Result and ELO-Goals, although using a different set of data including four European leagues and two international competitions.

**Table 3:** Statistical tests comparing the predictive qualities of different forecasting methods. The p-value compares each model to the model in the next row.

Forecasting Model	Average L_i_	Standard deviation L_i_	p-value (paired t-test)
Betting Odds	1.380	0.674	< 0.0001
ELO-Odds (k = 175)	1.391	0.706	< 0.0001
ELO-Goals (k_0_ = 4, λ = 1.6)	1.401	0.714	0.0202
ELO-Result (k = 14)	1.403	0.715	-

ELO-Goals being superior to ELO-Result confirms that the goal difference of a match contains more relevant information than its result (win, draw, lose). This is in line with similar results from [28] who showed that the average goal difference is a better measure for a team’s quality than the average points (both calculated over a number of matches).

The striking and novel result is the superiority of ELO-Odds to ELO-Goals which confirms that forecasts from previous matches are indeed useful in rating teams and a valuable source of information for forecasting future matches. Please note that this result is not notably depending on the choice of the parameter k as ELO-Odds is still outperforming ELO-Goals on a highly significant level (p < 0.01) if choosing extreme parameters like *k* = 30 or *k* = 400. Even for parameters like *k* = 20 or *k* = 500 ELO-Odds is still superior to ELO-Goals, but the difference is not significant anymore (see Table 4).

**Table 4:** Statistical tests comparing the predictive qualities of ELO-Odds (various extreme parameters) to ELO-Goals. The p-value compares each model to ELO-Goals.

Forecasting Model	Average L_i_	Standard deviation L_i_	p-value (paired t-test)
ELO-Odds (k = 175)	1.391	0.706	<0.0001
ELO-Odds (k = 400)	1.394	0.709	0.0044
ELO-Odds (k = 30)	1.396	0.707	0.0026
ELO-Odds (k = 500)	1.397	0.707	0.2118
ELO-Odds (k = 20)	1.398	0.714	0.0857
ELO-Goals (k_0_ = 4, λ = 1.6)	1.401	0.714	-

In fact, this shows that from a predictive perspective the betting odds known prior to a soccer match possess more information than the result known after the match. To put it simple, looking at the betting odds prior to a match gives you more relevant information on team quality and more valuable insights to performance analysis than studying the results afterwards. This result might partly be driven by the fact that the result of a match is a realization of the underlying probability distribution, while the betting odds represent this probability distribution. Including other match-related quality measures (besides results and goals) such as expected goals calculated from match statistics after a match could serve as basis for a useful additional ELO rating. Unfortunately, this would either require a publicly available source of expected goals covering the whole database or a database including comprehensive match statistics in order to calculate own measures of expected goals.

By design, we cannot expect the ELO-Odds model to provide better forecasts than the betting odds itself, as these are the only source of information for the model. Nevertheless, it is worth evaluating why there is such a clear gap in predictive qualities. Note that, although using betting odds as a source of information, the ELO-Odds model by far is exploiting less information than the betting odds. It can only extract team specific information from the betting odds and aggregate them in the ratings. Motivational aspects of a single match or any relevant information (like injuries or line-ups) that has become available in between two matches will not be reflected in ELO-Odds. Moreover, the actual result of the preceding match is not reflected in ELO-Odds, while it is surely influencing the betting odds. Finally, the ordered logit regression model using the ELO difference as single covariate might be a limiting factor, thus even an accurate rating does not necessarily lead to an accurate forecast.

### Analyzing individual team ratings

One important aspect of this study is to shed light on accurate (predictive) team ratings that are usually used as an intermediate result of forecasting models. Betting odds for a match can be seen as the market judgement for the quality of both teams participating. However, it is not straight forward to obtain a quantitative rating for each team from the betting odds of various matches. By using the betting odds as an input for the ELO calculation in ELO-Odds, we made the information included in the betting odds visible in terms of a team rating. The results of the previous section have already shown that ELO-Odds in general provides a superior estimation of team quality. We would like to illustrate this with reference to two remarkable examples. Certainly these examples cannot be seen as a proof for the superiority of ELO-Odds, but they can be useful to illustrate differences in quality estimation and how these can be used to understand the quality development of teams.

Before comparing ELO-Odds to ratings based on results or goals, we need to verify that the different ELO measures are comparable at all. Please note that due to the construction of the ELO calculation, points gained by one team are equally lost by another team. Therefore the sum of points for all teams in our database stays constant over the whole period of investigation. As a result, the ratings are comparable in terms of size and it is possible to compare the quality estimation of teams (in ELO points) between different models. In particular it becomes possible to analyze differences between ELO-Odds and ELO-Result on a team level and consequently to gain more detailed insights on the quality and performance development of each soccer team.

**Fig 5:**
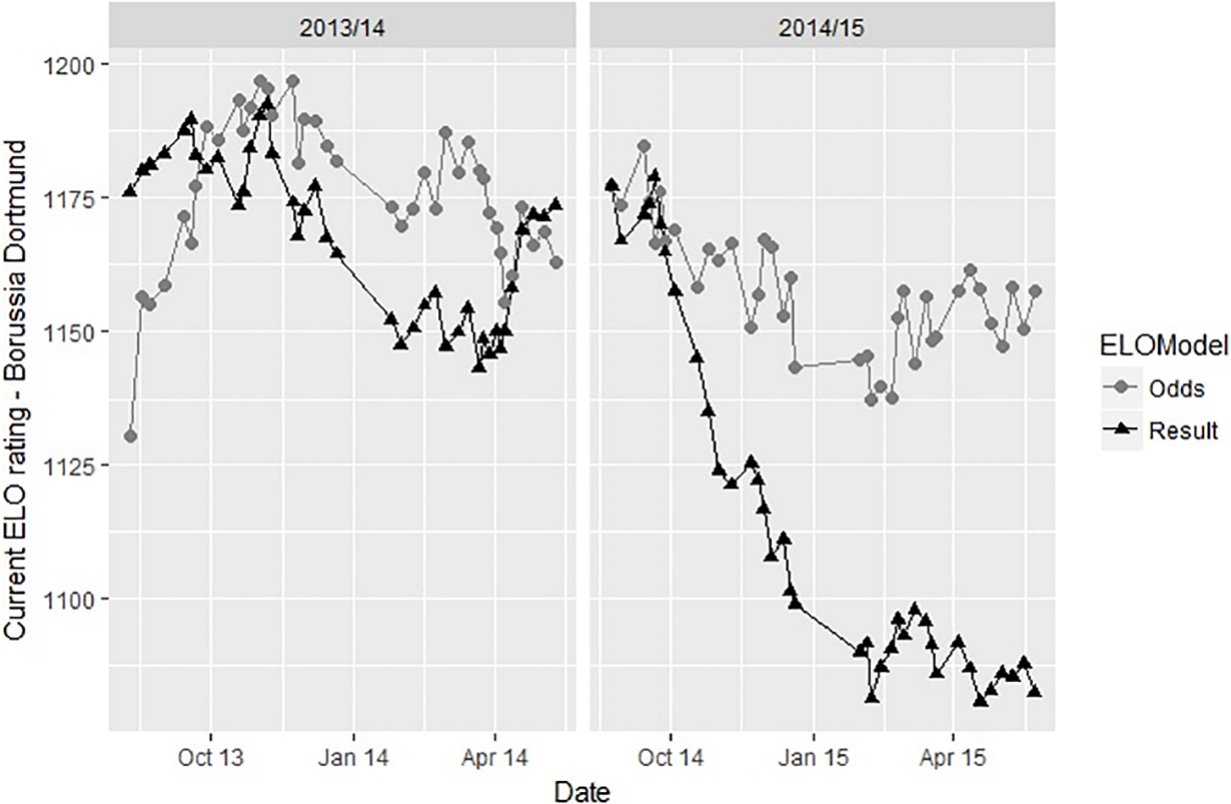
ELO-Odds and ELO-Result of Borussia Dortmund within the seasons 2013/14 and 2014/15.

Fig 5 shows the ratings for the German team Borussia Dortmund within the seasons 2013/2014 and 2014/2015 (period from August 2013 –May 2015). Both ELO-Results (*k* = 14) and ELO-Odds (*k* = 175) are presented. Having been one of the best teams in the previous seasons, Dortmund also finished successfully as 2^nd^ in the season 2013/2014. Despite small deviations (especially at the beginning of the season), the ratings for ELO-Result and ELO-Odds are mainly in line and virtually no difference in ratings exists at the end of the season. In February 2015 –after having massively unsuccessful results for half a year–Dortmund was in last position of the league table. Consequently ELO-Result shows a drastic decrease of almost 100 rating points. Surprisingly ELO-Odds for a long time hardly shows any reaction to the unsuccessful period, proving that the market judgement of the team quality was only weakly modified. The subsequent development might be interpreted as a confirmation of this judgement as Dortmund was playing a successful rest of the season and finished 2^nd^ and 3^rd^ in the two following seasons.

**Fig 6:**
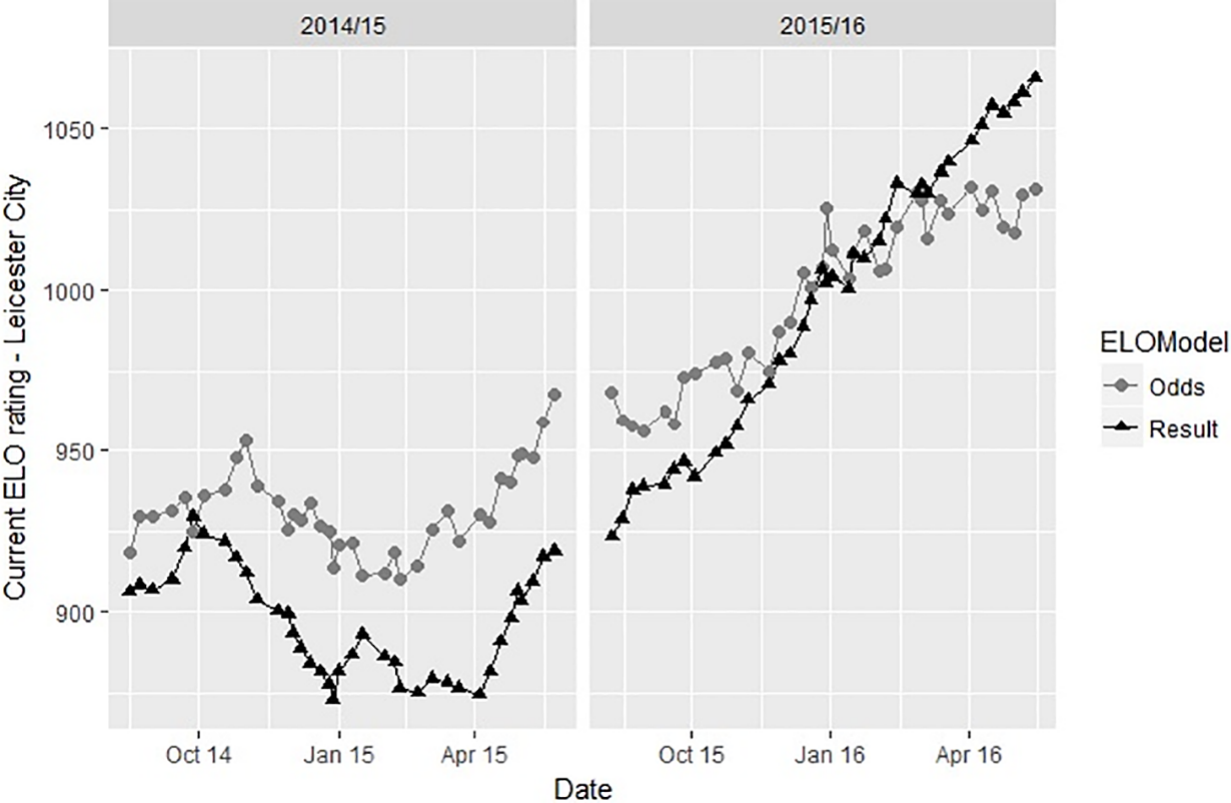
ELO-Odds and ELO-Result of Leicester City within the seasons 2014/15 and 2015/16.

Fig 6 shows the ratings for the English team Leicester City within the seasons 2014/2015 and 2015/2016 (period from August 2014 –May 2016). As a promoted team, Leicester finished 14^th^ in the 2014/2015 season. Throughout the complete season ELO-Odds is noticeably higher than ELO-Results. At the end of the season 2014/2015 there is a gap of roughly 50 points between the two ratings, indicating that the market clearly rated the team higher than the actual results revealed. During the season 2015/2016 Leicester won the Premier League being one of the most exceptional success stories in recent year’s association soccer. During that time ELO-Result increases dramatically, adding roughly 130 points to Leicester’s rating, whereas the increase in ELO-Odds is noticeably weaker (roughly 60 points). Similarly to the preceding example (yet in the opposite direction) the successful results were only mildly reflected in the market judgement on the team’s quality. Leicester finished 12^th^ in the following season, which again fits closer to the cautious market judgement than to the rating based on results.

In light of the results of this study, these examples show the effective use of a betting odds based rating in order to gain practical insights into the quality of soccer teams. Moreover, they are impressively showing that soccer results seem to be a very one-dimensional and thus an insufficient reflection of team quality. This result is in line with Heuer et al. [29] who describe “scoring goals” as a “highly random process”. This is the major reason for using hardly definable, but valuable criteria like chances for goals to estimate team quality [30]. Moreover, it gives rise to the idea of calculating advanced key performance indicators using position data from soccer matches [31,32].

Admittedly, the two examples refer to very special situations and were explicitly chosen in order to illustrate differences in ratings. Moreover, both situations were only discussed very briefly not considering events like the coach of Dortmund announcing to leave the club during the season or possible psychological and motivational effects hampering the performance of Leicester after the surprising championship.

### Analyzing league tables

Table 5 shows the final league table from the 2013/2014 season in Spanish Primera Division (left side). The usual perception would be that after 38 matches the teams are fairly well ordered related to their underlying quality throughout the whole season. As a comparison the teams were ordered following the average ELO-Odds rating during the season and presented at the right side of the table. There is a strong similarity between both rankings, but likewise there are a few notable discrepancies. Atletico Madrid won the title although clearly being ranked in third position by the betting market behind FC Barcelona and Real Madrid. Given the outstanding role of FC Barcelona and Real Madrid, this result might not be surprising and will be in line with the perception of many soccer experts, coaches and officials at that time. Differences concerning less successful teams are more interesting. According to the market valuation Levante UD was the worst team in the league during this season although finishing 10^th^ in the league table. In contrast to that, Betis Sevilla was ranked 11^th^ by the market, but in fact was relegated at the end of the season.

**Table 5:** Comparison between league table and average ELO-Odds rating (Primera Division 2013/14).

Pos.	Team	Goal Diff.	Points	Pos.	Team	ELO-Odds
1.	Atlético Madrid	51	90	1.	FC Barcelona	1294.59
2.	FC Barcelona	67	87	2.	Real Madrid	1229.22
3.	Real Madrid	66	87	3.	Atlético Madrid	1174.11
4.	Athletic Bilbao	27	70	4.	FC Valencia	1057.04
5.	FC Sevilla	17	63	5.	Athletic Bilbao	1045.58
6.	FC Villarreal	16	59	6.	Real Sociedad	1033.51
7.	Real Sociedad	7	59	7.	FC Villareal	1022.52
8.	FC Valencia	−2	49	8.	FC Sevilla	989.47
9.	Celta Vigo	−5	49	9.	Espanyol Barcelona	983.53
10.	Levante UD	−8	48	10.	FC Málaga	973.85
11.	FC Málaga	−7	45	11.	Betis Sevilla	968.70
12.	Rayo Vallecano	−34	43	12.	Celta Vigo	962.58
13.	FC Getafe	−19	42	13.	FC Getafe	946.62
14.	Espanyol Barcelona	−10	42	14.	FC Granada	941.67
15.	FC Granada	−24	41	15.	FC Elche	941.33
16.	FC Elche	−20	40	16.	Rayo Vallecano	940.17
17.	UD Almería	−28	40	17.	CA Osasuna	938.78
18.	CA Osasuna	−30	39	18.	Real Valladolid	933.25
19.	Real Valladolid	−22	36	19.	UD Almería	928.16
20.	Betis Sevilla	−42	25	20.	Levante UD	915.48

This comparison gives valuable insights to the difference between results and market valuation of teams. Certainly, we do not have full knowledge about the exact mechanisms of performance analysis in professional soccer clubs. From an outside position and following the detailed media coverage, however, it seems that results are by far the most important basis of decision-making. Under the background of this study, club officials should pay more attention to careful performance analysis by assessing various sources of information than solely looking at the results when evaluating the work of players and coaches.

### Betting returns

When investigating a quantitative model for forecasting soccer matches, a common approach is to examine the financial benefit of the model by back-testing various betting strategies and calculating the betting returns. For reasons of completeness and comparability to other studies, betting returns for different ELO models were calculated and can be found in S1 File. However, we would like to point out that gaining positive betting returns cannot be equated with a superior predictive quality of the underlying model as measured by statistical measures. The naïve model of assigning 100% winning probability to each away team would yield positive betting returns if the probability of away wins was generally underestimated in the betting odds. However, it would certainly not be judged as a valuable probabilistic forecasting model. This example illustrates that finding profitable betting strategies and finding accurate forecasting models are slightly different tasks.

In addition, ELO-Odds is intended to connect the advantages of betting odds and mathematical models by extracting information from betting odds and using them in mathematical models. Consequently it would–by design–be unreasonable to expect systematically positive betting returns from such a model. Based on these reasons, the focus of this study is on evaluating the predictive quality of a forecasting model in terms of statistical measures and its benefit in enabling insights to performance analysis.

## Discussion

Although the predictive power of betting odds is widely accepted [23,11], betting odds have not been used as a basis to create rankings and ratings. Lots of effort has been made in developing mathematical models in order to find profitable betting strategies and thus *beat* the betting market [1,20,16]. In contrast, we pursue the strategy of using betting odds as a source of information instead of trying to outperform them. As the results show, this is a promising approach in an attempt to extract relevant information that would be hardly exploitable otherwise in mathematical models.

**Fig 7:**
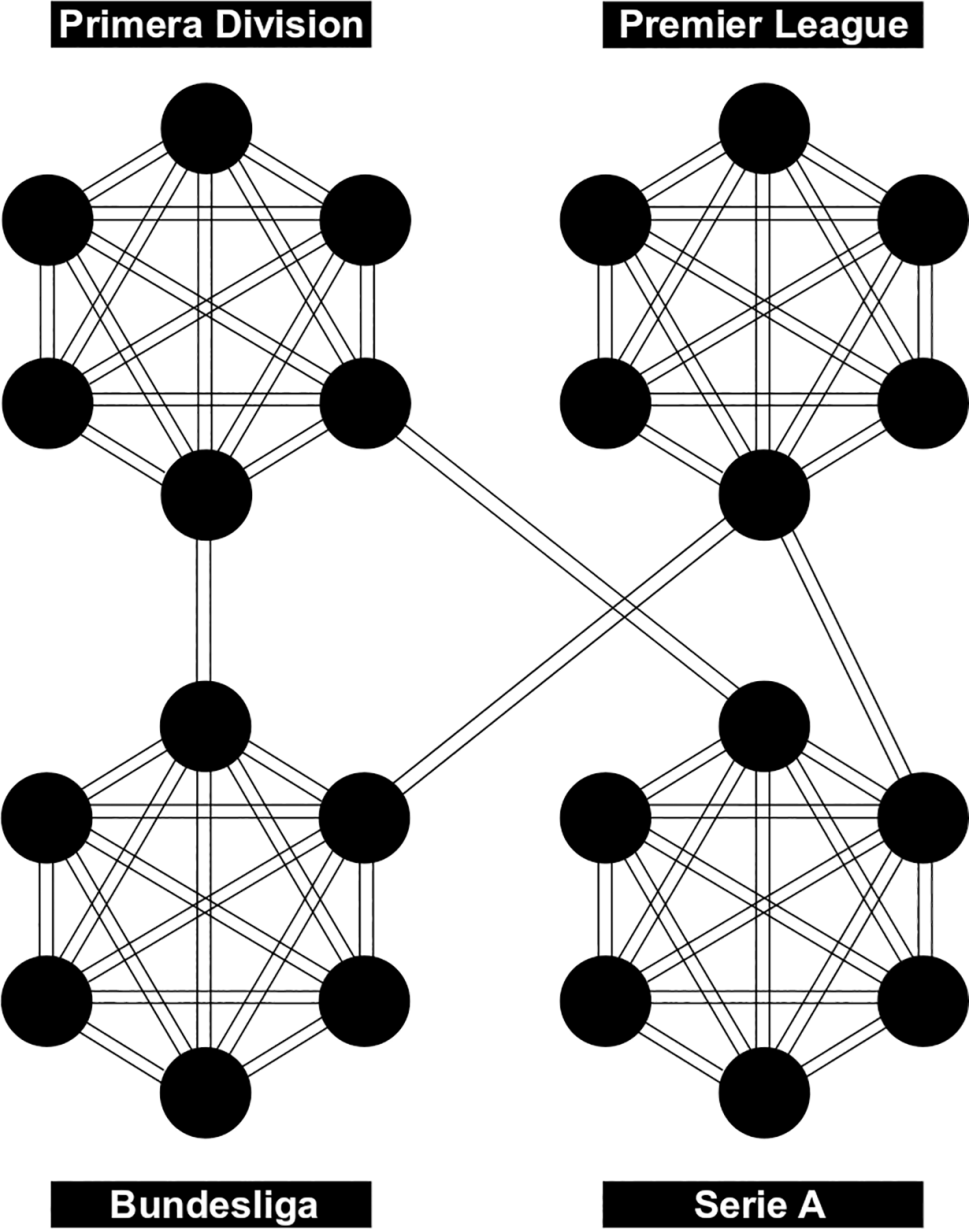
Simplified illustration of the database as a network of teams (nodes) and matches (edges).

We could successfully transfer prior results concerning ELO-ratings in association soccer [16] to a different set of data including both domestic and international matches. This transferability of results should not be taken for granted as the structure of the data heavily depends on the choice of teams and competitions. The data set used here is characterized by full sets of matches within the leagues and–in relation to this–only a few cross-references (i.e. international matches) between the leagues. See Fig 7 for a simplified illustration of the database as a network of teams (nodes) and matches (edges). Please note that for purposes of the presentation an explaining example is demonstrated, instead of the full database. The aforementioned study was missing international matches and different countries, but including lower leagues. Yet another situation applies for national teams who are playing relatively rarely. Tournaments as the World Cup take place only every four years and are played in a group stage and knockout matches. Further matches in continental championships or qualifications are lacking matches with opponents from different continents. In other sports or comparable contexts (such as social networks) the structure again might be completely different.

For data sets like the one used within this study, the ELO rating system might not be the optimal approach as it is not designed for indirect comparison. Each match directly influences the rating of both competitors and thus can indirectly influence the future rating of other teams. However, a match is never directly influencing the rating of a non-involved team. We would expect a notable benefit in treating teams and matches as a network and taking advantage of this structure for future rating approaches. It can be supposed that this will lead to a shortened time period to derive useful initial ratings and more accurate quality estimations, especially for teams not being part of cross-references (i.e. competing in an international competition) at all.

So far, only few attempts to make use of the network structure [33] or explicitly including indirect comparison [34] have been made in US College Football. Other methods like the Massey rating (see [35] for an introduction) can be argued to implicitly take advantage of the network structure. However, there is a lack of general theory and a theoretical framework that investigates the best rating methods for different types of network structures.

Another aspect contributes to the complexity of evaluating rating and forecasting methods. The quality of a rating and forecasting model such as ELO-Odds depends both on its ability in estimating team ratings and its ability to forecast the outcomes, given accurate ratings. As match results are affected by random factors, the true quality of a team is never known or directly observable and thus the quality of the rating can only be tested indirectly. Moreover, it can be assumed that the true quality of a team will be subject to changes over time. In view of this, it is difficult to prove which aspect of the model carries responsibility for achieving or not achieving a certain predictive quality.

To gain better insights into the quality of rating models, it will be useful to conduct further studies using a more theoretical framework. This could be achieved by constructing theoretical data sets including known team qualities (true ratings) and simulated data for the observable results, applying the rating models to this data set and then comparing the calculated ratings with the true ratings.

ELO-Odds provides clear evidence for the usefulness of incorporating expert judgement into quantitative sports forecasting models in order to profit from crowd wisdom. Further evidence for the power of expert judgement can be found in Peeters [20] where collective judgements on the market value of soccer players from a website are successfully used in forecasting tasks. Moreover, researchers recently have started attempts to extract crowd wisdom from social media data. An example aiming at soccer forecasting can be found in Brown et al. [36] where Twitter data are used to detect mispricing in live betting odds of the bet exchange Betfair.

## Conclusion

Within this study we made use of betting odds as a highly valuable tool in processing available information and forecasting sports events. The betting odds themselves are a measure for the expected success in the following match. Using our approach, we can directly map these expectations of the market to a quantitative rating of each team, i.e. a measure of team quality. This measure proves to be superior to results or goals when used within a framework of an ELO forecasting model. We did not evaluate the differences between ELO-Odds and the betting odds themselves in detail. Future studies investigating match related aspects (such as motivational aspects, line-up, etc.) might help to find and gain insights into factors that influence the betting odds of a match, but are not related to the general team quality. In contrast to prior research, we emphasized that rating methods and forecasting models can help to gain insights to the underlying processes in sports and that there is a strong link between forecasts and performance analysis.

The present study is further evidence that results and goals are not a sufficient information basis for rating soccer teams and forecasting the outcomes of soccer matches. Expert opinion can possess highly valuable information in forecasting, future rating and forecasting models should become more open to include sources of crowd wisdom into mathematical approaches. In times of social networks and online communication new possibilities have emerged and will keep emerging. Huge data sets from social media (e.g. Twitter data) or search engines (e.g. Google search queries) have just been started to be explored in the scientific community and are a challenging, but highly promising approach to be used in rating and forecasting. Due to the lack of an alternative, sport-scientific studies regularly use wins/losses, the number of goals or league table positions as a measure to differentiate between stronger and weaker soccer teams. With respect to the methods and results shown within this study, a measure based on betting odds would be more suitable than the aforementioned measures based on results, goals or league tables. This could be adapted in future research by taking advantage of the ELO-Odds rating as an improved method to assess team qualities.

## Supporting information

S1 File Appendix. Appendix including details on calculating probabilities from betting odds (Appendix A) and the investigation of betting strategies (Appendix B).
(DOCX)

S2 File MinimalDataSample. Data set including the minimal data needed to replicate the study as well as main results (ratings) intended to be usable by other researchers in future research.
(XLSX)
