# Supplementary material for: The Betting Odds Rating System: Using soccer forecasts to forecast soccer
Source: PLoS One. 2018 Jun 5;13(6):e0198668. doi: 10.1371/journal.pone.0198668 (PMC5988281; doi:10.1371/journal.pone.0198668)
Supplement: S1 File — Appendix including details on calculating probabilities from betting odds (Appendix A) and the investigation of betting strategies (Appendix B). (DOCX) [file pone.0198668.s001.docx]

**Appendix**

**Appendix A**

**Margins and calculating probabilities from betting odds**

Under the assumption that the bookmaker margin is distributed proportionately across all outcomes of a match, the probabilities can be obtained from the betting odds by basic normalization as follows:

Let ${odds}_{i}$ be the betting odds of outcome $i$ for outcomes $i=1\ldots n$ of a match (namely home win, draw and away win with regard to this study). Then the bookmaker overround $o$can be computed as

$$o=\sum_{i=1}^{n} {\frac{1}{odds}}_{i}$$

And the probability $p_{i}$ can be calculated as

$$p_{i}= \frac{1}{{odds}_{i ˙}o}$$

resulting in probabilities for each outcome summing up to 100%. The theoretical bookmaker payout is the reciprocal value of the overround. It is intended to present the percentual payout of the bookmaker, but has a theoretical character as this is only true in case the risks are perfectly balanced over all outcomes (i.e. the bookmaker pays out the same percentage of stakes no matter which outcome wins). Average overrounds and theoretical bookmaker payouts related to the database used within this study are shown in Table 1.

**Appendix B**

**Investigating betting strategies and betting returns**

The three ELO models (ELO-Result, ELO-Goals and ELO-Odds) were used to construct betting strategies and the betting returns are presented in Table 6. Let $p$ be the probability of a match outcome based on the forecasting model. Let $o$ be the decimal betting odds of the respective outcome. Like for any other analysis within this study, average betting odds were taken as a basis for the calculation of bet sizes and profits. Whenever the forecast indicated that the expected value of a bet was positive (i.e. $p\cdot o>1$), this bet was included into the calculation. From the known results of the matches, the payout of the bet was then calculated. Again we follow the methodology of Hvattum and Arntzen [16] and considered three different strategies to determine the stake of the bets. UNIT BET uses a stake of one unit for every bet placed. UNIT WIN uses a stake of $1/(o-1)$, that corresponds to a profit of one unit in case the bet wins. KELLY uses a stake of $(o\cdot p-1)/(o-1)$. The number of bets (#BETS) as well as the average bet size (BS) and the (averaged) total return on bets (TROB) are presented.

***Table 6: Results for various betting strategies based on different ELO models***

| Forecasting model | #BETS | UNIT BET | | UNIT WIN | | KELLY | |
| --- | --- | --- | --- | --- | --- | --- | --- |
|  |  | BS | TROB | BS | TROB | BS | TROB |
| ELO-Odds ($k=175$) | 6,562 | 1.000 | 0.913 | 0.701 | 0.954 | 0.047 | 0.930 |
| ELO-Goals ($k_{0}=4$, $\lambda=1.6$) | 7,179 | 1.000 | 0.912 | 0.685 | 0.940 | 0.068 | 0.943 |
| ELO-Result ($k=14$) | 7.381 | 1.000 | 0.888 | 0.688 | 0.935 | 0.071 | 0.932 |
